# mHealth to support resistance training using outdoor gyms: the *ecofit* hybrid type 3 implementation–effectiveness trial

**DOI:** 10.1093/tbm/ibag024

**Published:** 2026-05-01

**Authors:** Ronald C Plotnikoff, Anna K Jansson, Mitch J Duncan, Sam Beacroft, Adrian Bauman, John Attia, Emily R Cox, Jordan J Smith, Sara L Robards, Mark Babic, David R Lubans

**Affiliations:** Global Sport and Movement Collaborative, University of Newcastle, Callaghan, NSW, 2308, Australia; Global Sport and Movement Collaborative, Hunter Medical Research Institute, New Lambton Heights, NSW, 2305, Australia; School of Heath Sciences, University of Newcastle, Callaghan, NSW, 2308, Australia; Food and Nutrition Program, Hunter Medical Research Institute, New Lambton Heights, NSW, 2305, Australia; Global Sport and Movement Collaborative, University of Newcastle, Callaghan, NSW, 2308, Australia; Global Sport and Movement Collaborative, Hunter Medical Research Institute, New Lambton Heights, NSW, 2305, Australia; School of Medicine & Public Health, University of Newcastle, Callaghan, NSW, 2308, Australia; Global Sport and Movement Collaborative, University of Newcastle, Callaghan, NSW, 2308, Australia; Global Sport and Movement Collaborative, Hunter Medical Research Institute, New Lambton Heights, NSW, 2305, Australia; School of Public Health, University of Sydney, Camperdown, NSW, 2006, Australia; School of Medicine & Public Health, University of Newcastle, Callaghan, NSW, 2308, Australia; Global Sport and Movement Collaborative, University of Newcastle, Callaghan, NSW, 2308, Australia; Global Sport and Movement Collaborative, Hunter Medical Research Institute, New Lambton Heights, NSW, 2305, Australia; School of Exercise & Nutrition Sciences, Queensland University of Technology, Kelvin Grove, QLD, 4059, Australia; Global Sport and Movement Collaborative, University of Newcastle, Callaghan, NSW, 2308, Australia; Global Sport and Movement Collaborative, Hunter Medical Research Institute, New Lambton Heights, NSW, 2305, Australia; Global Sport and Movement Collaborative, University of Newcastle, Callaghan, NSW, 2308, Australia; Global Sport and Movement Collaborative, University of Newcastle, Callaghan, NSW, 2308, Australia; Global Sport and Movement Collaborative, Hunter Medical Research Institute, New Lambton Heights, NSW, 2305, Australia; Global Sport and Movement Collaborative, University of Newcastle, Callaghan, NSW, 2308, Australia; Global Sport and Movement Collaborative, Hunter Medical Research Institute, New Lambton Heights, NSW, 2305, Australia; Faculty of Sport and Health Sciences, University of Jyväskylä, Jyväskylä, Finland

**Keywords:** outdoor gym, resistance training, physical activity, implementation

## Abstract

**Background:**

There is a need to scale up effective physical activity interventions among the general population, particularly those incorporating resistance training. *Ecofit* is a community-based, multicomponent intervention promoting resistance and aerobic physical activity through smartphone technology, the outdoor built environment, and social support. This study aimed to scale up *ecofit* by comparing Low versus Moderate implementation support on the reach (outdoor gym use) of *ecofit* within two large regional municipalities.

**Methods:**

A hybrid type 3 implementation–effectiveness trial was conducted across two large municipalities in eastern Australia. Outdoor gyms (*n* = 18) were randomized to Low (ecofit app only) or Moderate [ecofit app, QR (quick response) codes on equipment, face-to-face workout sessions] implementation support. The primary outcome of “reach” was defined as the baseline-adjusted difference in the number of outdoor gym users (i.e. adults using outdoor gym equipment for resistance training) between groups. Reach was measured at baseline and 3-month follow-up using a modified *System for Observing Play and Recreation in Communities* tool, with blinded assessors observing community members perceived to be ≥18 years [categorized as Adults (aged 18–59) or Seniors (aged ≥60)]. Secondary outcomes included app uptake, dose received, implementation fidelity, and acceptability, feasibility, and dose-satisfaction regarding the app and guided sessions.

**Results:**

There was no significant difference in people using outdoor gym equipment for resistance training between Low and Moderate support groups at 3-month follow-up [incidence rate ratio (IRR) = 1.68, 95% CI: 0.96–2.94]. Among adults (aged 18–59), the Moderate support group showed significantly higher outdoor gym use at follow-up (IRR = 1.83, 95% CI: 1.01–3.31) compared to the Low support group. Over 6 months, 1273 users registered for the app, completing 503 workouts, 62% of which occurred indoors.

**Conclusions:**

*Ecofit* shows promise for promoting resistance training, particularly among adults. Broader marketing and enhanced engagement strategies may be required to increase outdoor gym use and sustain participation over time.

## Teaser

• This is the first physical activity implementation trial to focus on resistance training in the general population

• *Ecofit* shows promise for promoting resistance training using outdoor gyms

## Graphical abstract

**Graphical Abstract:**
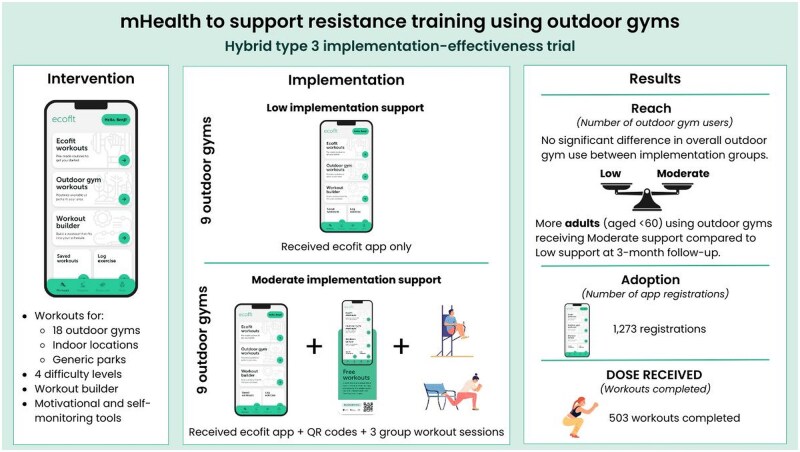

Implications

- **Practice:** mHealth may be better utilized as a supplement to more direct strategies for the promotion of outdoor gym use.
- **Policy:** Local governments should explore pairing outdoor gym installations with other support strategies to improve public engagement.
- **Research:** Future studies should develop strategies to promote sustained outdoor gym use and evaluate long-term health outcomes across diverse populations.

## Background

Physical inactivity is a leading cause of noncommunicable diseases worldwide and accounts for ∼9% of premature mortality [1]. Globally, nearly one-third of adults are classed as physically inactive [2], while 70%–90% of adults do not meet the resistance training (RT) guidelines [3–6]. To address the epidemic of physical inactivity, effective physical activity interventions, especially those targeting RT, need to be disseminated at scale.

Within physical activity research, there is momentum to adapt successful interventions tested under controlled conditions and deliver them on a larger scale, with the aim of providing benefit to the wider community [7, 8]. Where interventions do scale-up in the community, they often target aerobic activity only (e.g. 10 000 steps [9]). To our knowledge, no interventions targeting RT have been scaled up among the general population. It may be that interventions promoting RT are more complicated and expensive to scale up compared with those focusing on aerobic exercise, as RT interventions are often supervised, require specialized equipment (e.g. weight machines, free weights, elastic resistance bands), are delivered within exercise facilities, and require a different set of skills (e.g. correct lifting techniques versus going for a walk) [10, 11]. These barriers can be addressed through interventions that provide evidence-based resources to provide guidance, support, and motivation to complete RT across different settings.

One promising resource for increasing population physical activity levels is outdoor gyms, which have become a popular public health initiative among local governments and municipalities worldwide [12–14]. Outdoor gyms are broadly defined as simple, durable exercise facilities designed for RT, aerobic activity, balance exercise, and stretching, and operate without electricity [15–18]. Despite the widespread installation of outdoor gyms, evidence suggests that they are under-used by the public [18]. To maximize the potential of outdoor gyms, interventions to increase use need to be developed and evaluated.

Interventions that scale up from highly controlled settings (i.e. efficacy trials) toward dissemination in real-world conditions face challenges including community recruitment and sustainment [19, 20]. As a result, scaled-up interventions delivered in more “real-world” contexts are generally delivered with less fidelity and have smaller effects than their corresponding pre-scale-up trials [21], phenomena known as “program drift” and “voltage drop,” respectively [22]. This may limit the ultimate progress toward intervention sustainability and population impact. Thoughtful implementation support that enhances implementation and intervention fidelity and uptake may minimize “program drift” and “voltage drop” of efficacious and effective programs.

*Ecofit* is a community-based, multicomponent intervention that promotes resistance and aerobic physical activity using smartphone technology, the outdoor built environment (with a focus on outdoor gyms) and social support. The *ecofit* intervention has been evaluated in an efficacy trial targeting adults at risk of/diagnosed with type 2 diabetes [23], and in an effectiveness trial targeting inactive community-dwelling adults [24]. The efficacy trial demonstrated significant improvements in aerobic fitness and lower body muscular fitness at the 10-week primary timepoint [23]. The effectiveness trial showed significant improvements in upper and lower body muscular fitness at 9-month follow-up [24]. The primary aim of this study was to scale up *ecofit* to community implementation by comparing Low versus Moderate implementation support on the reach (outdoor gym use) of *ecofit* within two large regional municipalities in eastern Australia. Secondary implementation outcomes (uptake, dose received, fidelity, and acceptibility, feasibility and satisfaction of implementation strategies) and intervention outcomes (impact and acceptability, feasibility, satisfaction of the intervention) were also assessed.

## Methods

### Study design

A detailed account of the study protocol is presented elsewhere [25]. We conducted a hybrid type 3 implementation–effectiveness trial [26] involving 18 outdoor gym locations, across two local government municipalities (approx. population *n* = 391 000) in eastern Australia. Due to the constantly evolving terminology in implementation science, this study could also be referred to as a “hybrid effectiveness reach trial” according to work published following the protocol of this study [27]. In this study, the *ecofit* intervention was held constant, and outdoor gym locations were randomized to receive either (i) Low or (ii) Moderate implementation support. Baseline data collection for the primary outcome took place in April 2024 to avoid the winter season, where limited daylight in this part of Australia could impact these assessments. The intervention began in October 2024 and primary data collection occurred 3 months post the beginning of the intervention. Secondary outcomes were assessed at 3 and 6 months post the beginning of the intervention. Planning and design were guided by the Medical Research Council recommendations for developing and evaluating complex interventions [28] and the Consolidated Framework for Implementation Research [29]. The Standards for Reporting Implementation Studies (StaRI) initiative has been used in the reporting of this study [30]. The study received ethical approval from the university ethics committee (H-2018-0060).

### Recruitment, randomization, and setting

We developed a local media campaign using several strategies to promote *ecofit* across two local government municipalities in eastern Australia. Prior to the intervention, a large mailout delivered 64 700 postcards that promoted the *ecofit* mobile app to all households across the 75 suburbs surrounding all 18 outdoor gym locations included in the study catchment. An official media launch took place and involved local print and broadcast (i.e. local radio and television interviews) media. Full- and half-page advertisements were placed in a popular monthly local magazine with a reach of 67 000 weekly readers [31], alongside newsletters of consenting schools in the catchment area. An e*cofit* Facebook page was created to share promotional videos, visual advertisements, and information about physical activity.

Each outdoor gym was pair-matched according to area-level socioeconomic status (SES) (i.e. outdoor gyms in high-SES areas were matched with other high-SES outdoor gyms, and low-SES with low-SES). Outdoor gyms within each pair were then randomized to either the Low or Moderate implementation support condition. The randomization procedure was conducted by a researcher not part of the core research team, using an online randomization tool [32].

A detailed description of each outdoor gym included in this study can be found in Supplementary Material S1. “Pod-based” outdoor gyms comprised one station with ≥4 unique pieces of exercise equipment, while “trail-based” outdoor gyms consisted of ≥2 exercise stations placed along an existing walking path, where each station includes ≤3 unique pieces of exercise equipment. “Trail/pod-based” outdoor gyms included equipment stations placed along a walking path, with one or more stations including ≥4 unique pieces of exercise equipment [18].

We conducted a brief audit of the outdoor gyms included in the study. The audit tool is included in Supplementary Material S2, alongside audit results. Overall, the included outdoor gym locations were considered clean and safe with good accessibility; however, purpose-built lighting was limited and no locations had purpose-built shade.

### Intervention components, delivery, and implementation strategies

**Figure 1:**
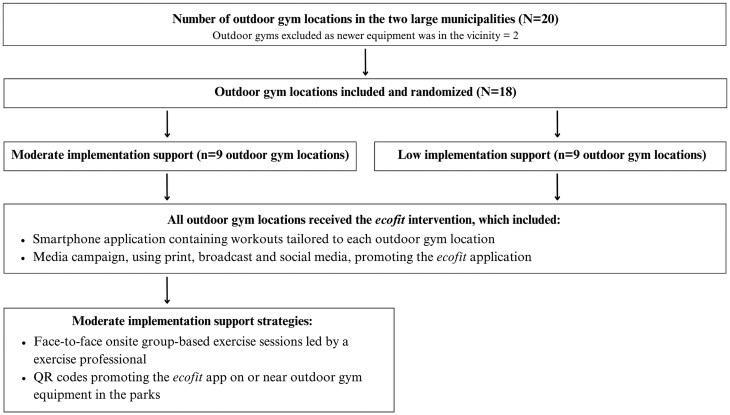
Outline of the Low and Moderate implementation support models.

For a detailed account of the intervention components, see the published protocol [25]. The Low and Moderate implementation support models are outlined in Fig. 1. Every outdoor gym location, along with surrounding suburbs, had access to the *ecofit* app. Outdoor gym locations assigned to the “Moderate support” condition further received (i) QR (quick response) codes placed on the equipment (linking to the app) and (ii) three face-to-face guided workout sessions delivered by an exercise professional.

### Intervention

#### Ecofit application

**Figure 2:**
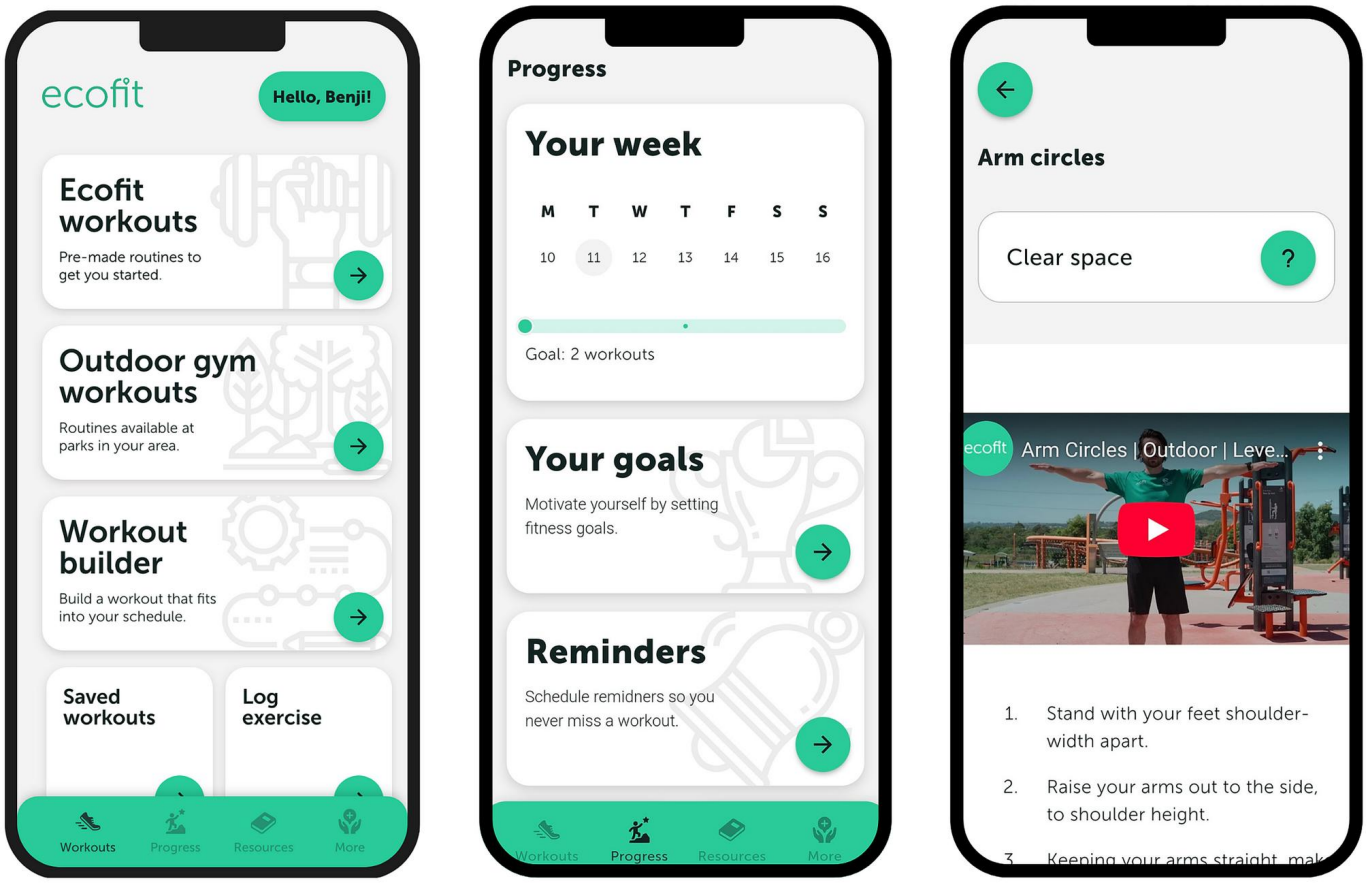
Screenshots of the *ecofit* app home screen and progress section.

The *ecofit* app could be accessed both via a smartphone app on Apple or Android devices, or as a web app via browsers. Figure 2 provides screenshots of the app home screen, “progress” section and instructions for one exercise. The app contained (i) videos of a real person demonstrating and explaining each exercise in an outdoor gym and indoor setting; (ii), written instructions for each exercise; (iii) predesigned workouts for outdoor gym, generic outdoor or indoor settings; (iv) four workout difficulty levels [i.e. beginner (Level 1), intermediate (Level 2), advanced (Level 3), pro (Level 4)]; (v) ability to build custom workouts based on location, equipment available, and body parts a person wants to focus on; (vi) self-monitoring functions (i.e. goal setting and self-assessment tool); (vii) an exercise library; and (viii) resources based on behavior change techniques [33]. When a user registered an *ecofit* account, they were shown an onboarding video that provided a clear overview of how to navigate the *ecofit* app. Users could see outdoor gym locations using an interactive map, or they could locate the address of each outdoor gym in the resources section.

#### Exercise settings and modalities

Predesigned workouts were created for outdoor gym, indoor, and generic outdoor (i.e. outdoor spaces such as parks that did not have outdoor gym equipment available) settings. They contained three upper body, three lower body, and two core exercises, with each exercise completed as two sets of 10 repetitions. Some “pro” (Level 4) workouts included three sets. Outdoor gym workouts were specifically designed to utilize the equipment at each outdoor gym location; workouts differed by location, depending on the available equipment. Generic outdoor workouts assumed that a user had access to a wall, bench, or railing. Indoor workouts assumed that users had a bench/chair and household items that could be used as weights (e.g. food cans, water bottles).

Predesigned workouts were classified as “Resistance,” “Integrated,” and “Trail,” with all workouts including eight resistance exercises. “Resistance” workouts included only resistance exercises and were designed for outdoor gym “pods,” generic outdoor workouts, and indoor workouts. “Integrated” workouts included aerobic activity (e.g. running, walking, stationary bike) interspersed throughout the eight resistance exercises and were designed for outdoor gym pods and generic outdoor workouts. “Trail” workouts were designed where outdoor gym equipment was spread along a trail. In these settings, aerobic activity was integrated into the workout when people moved between equipment stations.

### Implementation strategies (Moderate support)

In addition to receiving the *ecofit* app and general media campaign, outdoor gym locations randomized to the Moderate support group received two additional implementation support strategies. These implementation support strategies were guided by the findings from our previous studies [18, 23, 34, 35].

#### QR codes

QR codes were placed on and near equipment of outdoor gyms receiving Moderate implementation support. The QR code redirected people to the *ecofit* marketing website where they could find more information about *ecofit* and download the app. Websites that include program information and resources have been used in other larger scale physical activity interventions [9]. The QR code was located on a marketing sticker that contained a picture of the app home screen, along with the title “Free Workouts” (Supplementary Material S3).

#### Face-to-face guided workouts

Each outdoor gym receiving Moderate implementation support also hosted three face-to-face group-based workout sessions led by an exercise professional. The sessions were completed in the first month of the intervention, with two completed on weekdays and one on the weekend. In each session, the exercise professional demonstrated how to use the *ecofit* app by leading participants through the intermediate (Level 2) workout designed for that specific outdoor gym, while making modifications where necessary to account for participants’ capabilities. The sessions focused on demonstrating and correcting exercise techniques and answering any questions attendees had regarding the use of the *ecofit* app. The sessions were advertised with posters on or near outdoor gym equipment, posts on community social media pages for localities surrounding those outdoor gyms, and through notifications to app users who lived near outdoor gyms receiving the Moderate implementation support package.

### Changes to original protocol

After the original randomization of outdoor gym locations, two new outdoor gyms were added to the study. The two new locations were randomized and added to the intervention, increasing the number of outdoor gyms from 16 to 18. Due to scheduling issues with the postal services, the postcard mailout needed to be staggered over a 6-week timeframe, resulting in some suburbs receiving postcards 3 weeks prior to the official media launch. App metric data from these 3 weeks have been included in the analysis of secondary outcomes.

### Outcomes/measures and data collection

For a more detailed explanation of the outcomes, including measurement instruments, see the published protocol [25]. Guided by the evaluation roadmap for scaling-up physical activity and behavioral nutrition interventions [36], we selected reach as the primary outcome (i.e. reach), and also assessed a range of secondary implementation outcomes (uptake, dose received, fidelity, and acceptibility, feasibility and satisfaction of implementation strategies) and intervention outcomes (impact and acceptability, feasibility, satisfaction of the intervention). The primary implementation outcome of “reach,” described below, was measured at baseline and 3 months (primary timepoint). Table 1 presents the secondary outcomes and when they were collected.

**Table 1:** Secondary implementation outcomes and secondary intervention outcomes.

Outcome	Description	Measured at the following time-points			
		Baseline	3M	6M	Single timepoint
**Secondary implementation outcomes**					
**Uptake**	The total number of app registrations at 3 and 6 months post baseline		✓	✓	
** Dose received**	The number of participants who logged a workout, and the total logged workouts. Data relating to additional characteristics of “dose received” (exposure) included assessment of (i) changes in the workout difficulty by individuals, (ii) whether app users engage in different workout types, (iii) number of goals set and met, (iv) settings where the workouts were undertaken (i.e. outdoor gym, indoor, outdoor), and (v) The number of views of the resource material (i.e. videos and text) across the *ecofit* app at 3 and 6 months.		✓	✓	
**Uptake (Moderate support only)**	The number of scans of QR codes located on the outdoor gyms receiving Moderate implementation support. “Uptake” was also evaluated by the number of people who attended the in-person workout sessions at the outdoor gyms receiving Moderate implementation support. The exercise professionals conducting the in-person workout sessions collected count data on attendees, including perceived gender and age categories.		✓	✓	
**Fidelity of in-person session delivery**	Measured when a member of the research team observed each exercise professional on at least one occasion to assess how they conducted the workout sessions at the outdoor gyms receiving Moderate implementation support. Exercise professionals were required to deliver the following components: a warm-up, workout (e.g. provide instruction, give feedback, modify exercises, and cover all key muscle groups), cool-down and demonstrations of app use (Supplementary Material S4). The research member marked each component as “yes” (1) or “no” (0), with the total score ranging from 0 to 9.				Conducted during F2F sessions
**Acceptability, feasibility, and dose satisfaction of in-person sessions**	Participants who attended a face-to-face session completed as survey directly following the face–face sessions by scanning a QR code and completing online (Supplementary Material S5). The short survey included five questions with Likert responses (1 = strongly disagree to 5 = strongly agree). The two exercise professionals were interviewed at the conclusion of all in-person training sessions; perceptions about acceptability, feasibility and satisfaction of delivering the training sessions were explored.				At conclusion of each F2F session
**Implementation costs**	Cost of the general marketing strategy aimed at all areas surrounding outdoor gyms included in this study, in addition to the costs of the moderate implementation strategies.				Conclusion of study
**Secondary intervention outcomes**					
**Usage**			✓	✓	
**Impact**	“Impact” outcomes (i.e. upper and lower body strength) were collected via the built-in self-assessment feature in the *ecofit* app. Users could complete assessments of their upper and lower body strength at any time; their first assessment would be classed as “baseline.” The push-up and 60-s sit-to-stand tests have demonstrated strong and moderate validity in a sample of community-dwelling adults, respectively [39]. Impact is also considered to be a component of study effectiveness.	✓			No set timepoints
**Acceptability, feasibility, and dose satisfaction of the app**	Evaluated using a process evaluation survey through the app (Supplementary Material S6). Measures included satisfaction with the content (i.e. exercises, workouts, resources) and app usability. For each domain, users indicated their agreement with positively worded statements using a Likert scale (1 = strongly disagree to 5 = strongly agree).	✓			As users choose

#### Primary outcome

The primary outcome of “reach” was defined as the difference in the number of outdoor gym users (i.e. adults aged ≥18 using the outdoor gym equipment for RT activities) between the two implementation groups adjusted for baseline values. This was measured using a “modified version” of the *System for Observing Play and Recreation in Communities* [SOPARC Resistance Training (hereafter referred to as SOPARC-RT)] [18], which has been tested for interrater reliability [37]. SOPARC-RT was specifically adapted to capture additional data (not included in the original SOPARC protocol [38]) by coding the interaction with the outdoor gym equipment (i.e. “using equipment for RT,” “completing RT without equipment,” “stretching using equipment,” and “using equipment for aerobic exercise”) [37]. As a modification to the SOPARC-RT protocol, each user was counted once, regardless of how many times they returned to the observation area. See Supplementary Material S3 for the SOPARC-RT tool.

Baseline and follow-up (3 months) observations were conducted by trained research assistants who were blinded to outdoor gym randomization, over a 4-week period. Each outdoor gym was observed over 2 days (one weekday and one weekend day), with pair-matched outdoor gyms being observed concurrently. Each day was divided into three, 2-hr observation shifts: morning (6–8 a.m. on weekdays, 6.30–8.30 a.m. on weekends), midday (12–2 p.m.), and evening (4–6 p.m.) [18, 38]. At baseline and 3-month follow-up, each park was observed six times, totaling 108 (216 h) observations at each timepoint and 216 (432 h) in total. Only people perceived by the assessors to be over 18 years old were included; they were then categorized as Adults (aged 18–59) or Seniors (aged ≥60). Where sessions were postponed due to inclement weather, the observations were made up the following day or on the corresponding day of the following week.

### Statistical analysis

All analyses adhered to the intention-to-treat principle, including all randomized outdoor gyms. By design, there were no missing outcome data. Descriptive statistics were used to summarize baseline group characteristics. Between-group differences at follow-up were estimated using generalized linear mixed models, with fixed effects for the total baseline outdoor gym counts of the outcome and study group, and a random intercept for outdoor gym to account for clustering. Outcomes were total observation period counts, with six observation periods per outdoor gym at follow-up [40]. Model family and link functions were selected based on outcome distributions and residual diagnostics. Negative binomial models with a log link were used for counts of total users, females, males, and adults, while a logistic model was used for Seniors (≥1 user vs 0 user). No serious model assumption violations were observed. At follow-up, count outcomes are reported as marginal mean counts by group, with between-group differences shown as incidence rate ratios (IRRs). Dichotomous outcomes are presented as baseline-adjusted marginal predicted probabilities, with differences expressed as odds ratios (ORs).

To test the robustness of findings, three sensitivity analyses were conducted: (i) primary models were re-run with a fixed effect for outdoor gym quality; (ii) baseline and follow-up counts were aggregated per outdoor gym and analyzed using generalized linear models—originally planned as the primary analysis but later reclassified to preserve power; and (iii) mixed models estimated group differences over time using repeated measures, without conditioning on baseline. Family and link functions matched the primary analysis and were selected based on outcome distribution and diagnostics. Variation between outdoor gyms was estimated from a null model that only includes a random intercept for outdoor gym and an adjusted model including the model fixed effects. Variation between outdoor gyms is expressed as variance partition coefficients (VPC) negative binomial models [41, 42], and intraclass correlation in logistic models [43, 44]. All analysis were performed using Stata MP (version 17) and statistical significance was assessed at α = 0.05.

## Results

### Reach (primary outcome)

Table 2 shows the descriptive information for both groups and overall, at baseline. The number of clusters and outdoor gym SES were balanced across groups. There were differences between groups in the proportion of adults using RT equipment by gender and age at baseline. Descriptives for each of the study outcomes by group and time are presented in Table S1.

**Table 2:** Baseline characteristics for outdoor gym observations by group at baseline.

	Low support		Moderate support		Total	
	*N*	(%)	*N*	(%)	*N*	(%)
**Outdoor gym**	9		9		18	
**Outdoor gym SES**						
** SEIFA value 1–5 (low)**	4	(44.4)	4	(44.4)	8	(44.4)
** SEIFA value 6–10 (high)**	5	(55.6)	5	(55.6)	10	(55.6)
Total users						
** Perceived sex**						
** Male**						
** Total counts**	155		77		232	
** Mean (SD)/Outdoor gym**	17.22	(9.07)	8.56	(6.41)	12.89	(8.82)
** Median (IQR)/Outdoor gym**	20	(11.0–24.0)	8	(3.0–14.0)	12	(6.0–20.0)
** Female**						
** Total counts**	85		29		114	
** Mean (SD)/Outdoor gym**	9.44	(6.33)	3.22	(2.86)	6.33	(5.74)
** Median (IQR)/Outdoor gym**	11	(4.0–15.0)	3	(2.0–4.0)	4	(2.0–11.0)
** Perceived age**						
** Adult (18–59)**						
** Total counts**	208		87		295	
** Mean (SD)/Outdoor gym**	23.11	(13.44)	9.67	(6.52)	16.39	(12.36)
** Median (IQR)/Outdoor gym**	33	(12.0–33.0)	9	(4.0–14.0)	13	(6.0–33.0)
** Senior (≥60)**						
** Total counts**	33		20		53	
** Mean (SD)/Outdoor gym**	3.67	(4.27)	2.22	(2.73)	2.94	(3.56)
** Median (IQR)/Outdoor gym**	2	5.0)	1	(0.0–3.0)	2	(0.0–5.0)
**Performed RT using equipment**						
** Total counts**	207		87		294	
** Mean (SD)/Outdoor gym**	23.00	(11.77)	9.67	(6.89)	16.33	(11.60)
** Median (IQR)/Outdoor gym**	27	(13.0–31.0)	8	(4.0–17.0)	15	(6.0–27.0)

Gender and age counts reflect observed person irrespective of type of interaction with equipment/activity.

### Primary outcome (reach)

At baseline, 207 individuals were observed using outdoor gym equipment to perform RT at outdoor gyms receiving Low implementation support (Adults = 179; Seniors = 28), compared with 87 individuals at sites receiving Moderate support (Adults = 71; Seniors = 16). At the 3-month follow-up, 156 individuals were observed using the equipment to perform RT at outdoor gyms receiving Low-support (Adults = 124; Seniors = 32), and 74 individuals at sites receiving Moderate support (Adults = 65; Seniors = 9).

Table 3 presents baseline-adjusted marginal means at follow-up and between-group differences for each outcome. Although not statistically significant, the Moderate support group had more total users (IRR = 1.68, 95% CI: 0.96–2.94), females (IRR = 1.90, 95% CI: 0.58–6.18), and males (IRR = 1.05, 95% CI: 0.63–1.75) at 3 months compared with the Low support group. For Adults (aged 18–59), there was a significantly higher number of users in the Moderate support group at 3-month follow-up compared to the Low support group (IRR = 1.83, 95% CI: 1.01–3.31). The proportion of outdoor gyms with Senior (aged ≥60) users was lower in the Moderate group relative to the Low support group at follow-up, but this difference was not statistically significant (OR = 0.44, 95% CI: 0.13–1.50). The adjusted VPC was markedly lower than the unadjusted VPC indicating that between-outdoor gym variation was largely explained by the baseline values of the outcome and intervention group.

**Table 3:** Baseline adjusted between group differences at follow-up.

	Low	Moderate	Low	Moderate	Between group difference		
	Clusters (Obs)	Clusters (Obs)	*M* (95% CI)	*M* (95% CI)	IRR	VPC Adj.	VPC
**Overall use**	9 (6)	9 (6)	1.90 (1.45–2.48)	3.19 (1.98–5.14)	1.68 (0.96–2.94)	<0.01	0.51
**Sex**							
** Females**	9 (6)	9 (6)	0.67 (0.34–1.29)	1.27 (0.41–3.91)	1.90 (0.58–6.18)	0.26	0.50
** Males**	9 (6)	9 (6)	1.40 (1.08–1.82)	1.48 (0.97–2.25)	1.05 (0.63–1.75)	<0.01	0.37
**Age group**							
** Adults (18–59)**	9 (6)	9 (6)	1.52 (1.13–2.03)	2.77 (1.67–4.60)	1.83 (1.01–3.31)	0.02	0.49

			Pred. probability (95% CI)	Pred. probability (95% CI)	OR (95% CI)	ICC Adj	ICC
** Seniors (≥60)**	9 (6)	9 (6)	0.28 (0.17–0.42)	0.16 (0.08–0.30)	0.44 (0.13–1.50)	0.10	0.38

*M* (95% CI) at follow-up are the marginal mean counts, except for Seniors which report model-adjusted predicted probabilities (i.e. the estimated probability that an outdoor gym had any senior users). Between group difference in the baseline-adjusted follow-up counts (e.g. Moderate/Low). OR is the odds of any seniors observed at follow-up adjusted for baseline. VPC Adj./ICC Adj. are based on model including fixed effects for group, baseline value of the outcome (outdoor gym level) and random intercept for outdoor gym. Clusters (Obs) refers to the number of unique outdoor gyms and total observation periods contributing to estimates. ICC, intraclass correlation; Diff., difference; Pred., predicted.

Sensitivity analyses adjusting for outdoor gym quality (Table S2) and aggregating outcomes at the outdoor gym level (Table S3) yielded similar results to the primary analysis in both the direction and magnitude of effects. However, the difference in Adult (aged 18–59) users between groups was no longer statistically significant when adjusting for outdoor gym quality (IRR = 1.81, 95% CI: 1.00–3.31). Further sensitivity analyses comparing within- and between-group changes from baseline (Table S4) showed consistent directional effects but typically underestimated effect sizes. This pattern likely reflects baseline imbalances between groups, combined with opposing pre–post changes, with the Low support group generally declining and the Moderate support group showing improvement.

### Secondary implementation outcomes

Table 4 presents results regarding “uptake” and “dose received.” A more detailed account of “dose received” can be found in Table S5.

**Table 4:** Uptake and dose received of the *ecofit* app.

Metric of app use	3M (primary endpoint)	3–6M	Total (baseline–6M)
**Uptake**			
** QR scans (Moderate implementation)**	141	123	264
** Registrations**	1161	112	1273
Dose received			
** Total predesigned workouts completed**	362 (62 users)	141 (19 users)	503 (69 users)
** Indoor**	223 (39 users)	89 (13 users)	312 (45 users)
** Outdoor generic**	108 (20 users)	40 (8 users)	148 (22 users)
** Outdoor gym**	31 (19 users)	12 (6 users)	43 (22 users)
** Users completing >1 workouts**	32	15	36
** Total workouts started**	824 (159 users)	252 (40 users)	1076 (185 users)
** Number of goals set**	61 (50 users)	9 (8 users)	70 (57 users)
** Mean workouts completed by user, mean ± SD**	7.29 ± 14.7	7.42 ± 9.38	7.29 ± 14.74
** Mean indoor**	5.72 ± 9.35	6.85 ± 8.26	6.93 ± 12.2
** Mean outdoor**	5.4 ± 7.1	5 ± 5.4	6.73 ± 9.79
** Mean outdoor gym**	1.63 ± 1.12	2 ± 1.55	1.95 ± 2.08

#### Uptake

At the primary endpoint (3 months), 1161 people had registered an account on the *ecofit* app. At the 6-month timepoint, a further 112 people had registered an *ecofit* account.

#### Dose received

At the 3-month primary timepoint, 362 workouts had been completed by 62 unique users, with 36 of these users completing multiple workouts. Indoor workouts were most popular (*n* = 223), followed by generic outdoor workouts (*n* = 108) and outdoor gym workouts (*n* = 31). Between the 3- and 6-month timepoints, a further 141 workouts in total were completed by 19 users.

Full data for the following results can be found in Table S5. At the primary timepoint, “beginner” was the most popular workout level (*n* = 202), followed by “intermediate” (*n* = 95), “advanced” (*n* = 51), and “pro” (*n* = 14) workouts. A small number of users changed their workout level (*n* = 19) at the primary timepoint, increasing by one, (*n* = 10), two (*n* = 6), or three (*n* = 3) levels. At the primary timepoint, 55 custom workouts had been completed by 16 unique users, while 85 workouts were completed by nine users up to the secondary timepoint. “Intermediate” custom workouts were most common at the primary (*n* = 40) and secondary timepoints (*n* = 42). Goal setting was utilized by 50 users, who set a combined 61 goals by the primary timepoint. At the secondary timepoint, 8 users had set a combined 9 goals.

At the primary timepoint, 13 users had completed workouts in multiple settings, with users completing indoor and generic outdoor workouts (*n* = 8), generic outdoor and outdoor gym workouts (*n* = 2) or completing workouts across indoor, generic outdoor and outdoor gym settings (*n* = 3). Of the 108 generic outdoor workouts, users completed more resistance workouts (*n* = 62) than integrated workouts (*n* = 46). Most of the completed outdoor gym workouts were trail-based (*n* = 21), followed by resistance (*n* = 8) and integrated (*n* = 2) workouts.

Across the intervention, workout videos were viewed 3638 times, with most views occurring up to the primary timepoint (81%, *n* = 2932 views). The number of unique viewers could not be obtained.

#### Uptake (QR codes—Moderate implementation support)

QR codes were placed on outdoor gyms receiving moderate implementation support. At the 3-month primary timepoint, 141 people had scanned the QR codes. Between the primary and secondary timepoints, a further 123 people scanned the QR codes.

#### Uptake (moderate implementation support)

A total of 26 people expressed interest and 14 people attended the face-to-face workout sessions (*n* = 27 sessions in total) (Table S6). Among session attendees, exercise professionals running the sessions identified eight females [three adults (18–59 years), five seniors (≥60 years)] and six males (three adults, three seniors).

#### Fidelity of in-person session delivery

A total of three face-to-face sessions were observed. The sessions were delivered as intended, with instructors consistently adhering to the session format. Observations from three sessions yielded a mean fidelity score of 8.7/9.

#### Acceptability, feasibility, and dose satisfaction of face-to-face sessions (moderate implementation support)

Of the 11 face-to-face session attendees who completed a feedback survey, all attendees were satisfied with the in-person sessions (mean score 5/5) and believed that their instructor was knowledgeable about RT and physical activity (mean 5/5). Attendees believed the session improved their confidence [mean 4.64/5 (SD = 0.5)] and technique [mean 4.55/5 (SD = 0.52)] to undertake RT and felt that the session was appropriate for their needs [mean 4.73/5 (SD = 0.47)]. All respondents agreed or strongly agreed with each statement. Full results can be found in Table S6.

The two exercise professionals who delivered the in-person sessions were interviewed following completion of the sessions (Supplementary Material S8). The exercise professionals found the sessions to be acceptable and feasible, noting that sessions were suitable for all participants as they were an appropriate length and could be easily modified to suit participant ability. The exercise professionals were satisfied with how the sessions were organized by the research team, giving them confidence to deliver the sessions smoothly. Exercise professional feedback is provided in Supplementary Material S7.

#### Implementation costs

Full costs of the implementation strategies can be found in Table S7. The general marketing campaign targeted at both implementation support groups totaled AUD $25 869. Moderate implementation strategies totaled AUD $1166 for the stickers placed on equipment and AUD $4500 for the in-person sessions.

### Secondary intervention outcomes

#### Impact

Baseline self-assessments were completed by 32 *ecofit* users. Of this group, two users completed a follow-up assessment. Therefore, an analysis of intervention impact/effectiveness could not be completed due to the small sample size.

#### Acceptability, feasibility, and dose satisfaction of the app

A total of 38 app users completed the user experience survey (Table S6). Overall, 79% of users agreed or strongly agreed that the *ecofit* app was satisfying to use, and 82% agreed it easy to navigate. Fewer users agreed that the app provided sufficient information to perform RT using outdoor gyms (55%), motivated them to use outdoor gyms more frequently (60%), or increased their confidence to use outdoor gyms (41%).

## Discussion

This study evaluated the community-wide implementation of the *ecofit* intervention by testing a Low and Moderate implementation support model in two large regional municipalities in eastern Australia. This appears to be the first community-based physical activity intervention targeting RT to be scaled up. In the primary analysis, there was no significant difference in reach between outdoor gyms in the Low and Moderate support groups when adjusted for baseline. Subgroup analyses however revealed that a significantly greater number of adults (aged 18–59 years) were using outdoor gyms receiving Moderate support compared to those receiving Low support at follow-up. These results make an important addition to the physical activity implementation literature, particularly concerning mHealth and RT.

We did not observe significant differences in outdoor gym use within or between parks receiving Low and Moderate implementation support, suggesting that the Moderate implementation strategies of face-to-face guided workouts and stickers with QR codes were not broadly effective at increasing use among the general population. However, the significantly greater number of adults using parks in the Moderate group compared to the Low group is an encouraging finding, indicating that the implementation strategies may have been more effective for those under the age of 60 than for Seniors. This may reflect greater familiarity and confidence with using technology [45], such as scanning QR codes to access app-based resources among these younger adults.

At follow-up, overall outdoor gym use declined across both implementation groups compared with baseline. This may have occurred because the follow-up observations took place during summer, whereas baseline observations were conducted in autumn. High summer temperatures (see Table S9 for recorded temperatures on observation dates) and ultraviolet light exposure [46], combined with the lack of shade at outdoor gyms, may have discouraged use. Shade provision has previously been identified as an important enabler of outdoor gym participation [12, 35, 47, 48].

Outdoor gym randomization may have impacted the primary outcome of reach, with baseline observations revealing differences between groups. By chance, the outdoor gyms receiving Low implementation support were generally located in busier/convenient locations with nice scenery, quality greenspace and good amenities, while outdoor gyms receiving Moderate support were often located in quieter suburban locations. If outdoor gyms in busier/convenient locations had received Moderate implementation support, the impact of the Moderate implementation strategies may have been more pronounced. These contextual and environmental variations highlight the importance of considering outdoor gym characteristics, alongside SES during study group allocation, and suggest that future trials utilizing existing infrastructure may benefit from adjusting the randomization process to account for general location usage and resources on offer in addition to outdoor gym SES.

Most predesigned workouts completed by users were indoor or generic outdoor workouts, rather than outdoor gym workouts. There are several potential reasons for this result. Most of the workouts completed were “beginner” and “intermediate” levels, suggesting that users completing these entry-level workouts may have been newer to RT and/or experienced lower exercise self-efficacy and may have preferred to exercise in private settings rather than outdoor gyms, reducing the possibility of judgment and scrutiny [49]. Additionally, while app users who completed the acceptability survey reported increased motivation and confidence to use outdoor gyms, the small sample size and potential selection bias suggest that these findings may not reflect broader app user sentiment. As previously highlighted, seasonal weather may have also contributed to the low use of outdoor gym equipment [46]. With outdoor gyms having no shade for daytime workouts and limited lighting for evening workouts, participants may have opted for indoor or shaded environments. Participants may have also preferred the convenience and time efficiency of indoor workouts, which eliminated barriers such as travel to outdoor gym locations [50]. The popularity of indoor workouts is an encouraging finding, demonstrating the usability of the app across other settings; however, it may have reduced uptake of outdoor gym workouts.

Attendance to the in-person exercise sessions hosted by outdoor gyms receiving Moderate implementation support was poor. The likely predominant reason for the poor attendance is the way sessions were advertised i.e. (i) posters on equipment, (ii) posts in community Facebook groups, and (iii) notifications to selected app users. Due to the intervention design, where the Moderate and Low implementation support strategies were being compared, advertising methods with broader reach (e.g. social media, television, radio) were not used to limit contamination between implementation support groups. This increased the likelihood that only people who attended or lived near outdoor gyms receiving Moderate implementation support were informed about the sessions. In addition, weather conditions during multiple sessions were poor, with financial and time constraints restricting the research team’s ability to reschedule sessions (Table S8). Despite the poor attendance, these sessions were well received, with participants giving favorable feedback across all domains. In-person sessions remain a potentially beneficial implementation strategy if they can be advertised more effectively.

This study observed a large discrepancy between “uptake” (i.e. number of registrations) and “dose received” (i.e. number of users completing workouts). This is consistent with previous physical activity research where individuals often report intent to change behavior but do not take action [51]. User attrition in studies of this nature has been previously examined in the mHealth literature. Interventions focused on aerobic physical activity employing ecological recruitment strategies (i.e. spontaneous user registration) have consistently reported higher attrition rates and lower levels of user engagement compared to trials using active recruitment strategies [52, 53]. For example, at 12-month follow-up, Vandelanotte and colleagues observed 5.7% retention among users of the 10 000 steps intervention, while Wanner and researchers observed a retention rate of 36.4% among users recruited spontaneously [54, 55]. A potential explanation for the larger than expected gap was the focus on RT rather than aerobic PA. Although our study aimed to address multiple barriers to RT participation (i.e. cost, equipment, instruction), people may still have been hesitant to participate due to a lack of skill, knowledge, and/or self-efficacy to complete RT [10, 11]. A further reason for the incongruence between app registration and its uptake is that this trial did not include social support and accountability features (such as group exercise and in-person assessments) reported in previous efficacy and effectiveness trials [54, 55]. Additionally, users had the ability to view an entire workout including instructional videos without pressing “start,” therefore not logging a workout. Moreover, if a person pressed start, they could view the entire workout and videos without formally progressing through the workout. These features are not necessarily limitations of the app and may encourage app use; however, they may explain a proportion of the incomplete workouts and the limited reported data regarding app usage. Although a primary purpose of the *ecofit* app was to equip people with the skills and motivation to initiate behavior change (sustained app use is not necessary for behavior change maintenance [56]), the large proportion of non- or single-use participants suggests that initial engagement may still need to be strengthened.

Research assessing the drop-off in mHealth use has previously recommended the integration of behavior change techniques and a focus on quality user experience (e.g. ease of use, aesthetic design) [57, 58]. The design and content of the *ecofit* app were grounded in established, evidence-based theoretical frameworks informed by extensive user input from prior studies [29, 50, 59]. The app incorporated multiple features based on behavior change techniques commonly cited in the mHealth literature, such as goal setting, self-monitoring, behavioral instruction, and education features [60]. Feedback from users who completed the user experience survey in our study indicated high levels of satisfaction with the app’s design and functionality, with many finding it easy to navigate and reporting that it increased their motivation to exercise using outdoor gyms. However, engagement with these features ultimately depended on users’ initial motivation to interact with the app, as automatic and external notifications from the research team were intentionally omitted to avoid overwhelming or disengaging users. Future studies should consider incorporating a balanced notification protocol that encourages greater initial user engagement and adherence, without overwhelming participants [61, 62].

Public health campaigns promoting behavior change typically need to be both intense and sustained to achieve even modest changes at the population level [63]. The localized media campaign for this trial was not large or sustained enough to generate the level of uptake required to influence physical activity behavior on a whole population level. Initial observation indicated that the postcards did not make a significant contribution to the uptake of *ecofit*. While the media launch produced an initial increase in uptake, this was not maintained due to limited follow-up media activity. A more comprehensive social media strategy, including the use of paid social media advertisements, may have achieved greater uptake.

### Strengths and limitations and future directions

To our knowledge, this is the first scaled-up physical activity intervention that utilized mHealth technology to promote RT. A strength of the study is the use of the SOPARC-RT instrument to assess outdoor gym use. SOPARC-RT was adapted from the validated SOPARC tool and has demonstrated good interrater reliability [37]. Another strength was the quality of the *ecofit* app, which is user friendly, easy to navigate, and aesthetically pleasing. However, there are some limitations that should be noted. The SOPARC-RT observations were conducted for three, 2-hr time-periods (totaling 12 hr for each location) across one weekday and one weekend day and did not capture usage outside of these times. Financial constraints meant that including longer surveillance periods was not possible. Additionally, the methods of advertising face-to-face sessions for parks receiving moderate implementation support may have led to contamination between the groups, with people living near parks receiving Low support still attending these sessions. Another limitation was the app was relatively static with limited personalization. Users were not reminded to increase workout difficulty and there was no reward for workout completion or progression (e.g. badges, user levels). Such features may have increased sustained usage and engagement; however, cost logistics meant that these features could not be incorporated into the app. Notifications to use the app were not sent out throughout the intervention which may have contributed to the limited app use. However, with people able to turn notifications off, the impact of semi-regular notifications is unknown. The lack of impact data from the self-assessments is also a limitation, as we could not obtain relevant effectiveness data. Future research should consider additional or more intensive implementation strategies, particularly those engaging local councils and community agencies, to increase outdoor gym use among both adults under 60 years and Seniors.

## Conclusion

*Ecofit* is a potentially valuable tool for promoting RT across the adult population. The current app is a culmination of multiple research studies [23, 24, 64] and has been designed with strong consumer input. *Ecofit* may also be a useful tool for health clinicians such as general practitioners and exercise physiologists who require a cost-free, evidence-based resource to provide to people in their care. Among the general population, *ecofit* would benefit from more sustained, wider-reaching marketing, including social media. A potential strategy to improve the reach and uptake of *ecofit* is through partnerships with more local councils interested in increasing outdoor gym use. Adding more outdoor gym locations, while making use of council media reach, may improve *ecofit* uptake across a diverse range of settings. Developing strategies to increase attendance and feasibility of face-to-face sessions must also be considered.

## Supplementary Material

ibag024_Supplementary_Data

## Data Availability

De-identified data from this study are not available in a public archive. De-identified data from this study will be made available (as allowable according to institutional IRB standards) by emailing the corresponding author.

## Welfare of animals

This article does not contain any studies with animals performed by any of the authors.

## Transparency statement

- Study registration: This trial was preregistered with the Australian and New Zealand Clinical Trial Registry (ACTRN12624000261516).
- Analytic plan pre-registration: The analysis plan was not formally pre-registered; however, it can be found in the published protocol.
- Analytic code availability: Analytic codes used to conduct the analyses presented in this study are not available in a public archive. They may be available by emailing the corresponding author.
- Materials availability: All materials used to conduct the study are available in the Supplementary Material.
