# Supplementary material for: mHealth to support resistance training using outdoor gyms: the ecofit hybrid type 3 implementation–effectiveness trial
Source: Transl Behav Med. 2026 May 1;16(1):ibag024. doi: 10.1093/tbm/ibag024 (PMC13134382; doi:10.1093/tbm/ibag024)
Supplement: ibag024_Supplementary_Data [file ibag024_supplementary_data.zip › Supplementary material 8. Fitness professional feedback.docx]

**Supplementary material 8. Fitness professional feedback**

**Fitness professional 1**

**Acceptable/suitable**

The ecofit f2f sessions were acceptable (suitable) for the people that attended.

1. Strongly disagree, 2 – disagree, 3 – neutral, 4 – agree, 5 – strongly agree

Comments: The ecofit f2f sessions were highly suitable for all participants that attended.

**Feasible**

The ecofit f2f sessions were feasible (they were convenient and a good use of time)

1. Strongly disagree, 2 – disagree, 3 – neutral, 4 – agree, 5 – strongly agree

Comments: delivering the intervention felt like a highly valuable use of time – it provided participants the opportunity to be guided not only in exercise performance, but also on how to use the app correctly and effectively.

Finding a time of the day to hold f2f sessions that suit both trainer and as many participants as possible may be the only feasibility issue.

**Satisfaction**

I was satisfied with my delivery of the ecofit f2f sessions.

1. Strongly disagree, 2 – disagree, 3 – neutral, 4 – agree, 5 – strongly agree

Comments: the project team made delivery of the intervention easy to conduct and the app interface was very easy to navigate and explain to participants.

**Fitness professional 2**

**Acceptable/suitable**

The ecofit f2f sessions were acceptable (suitable) for the people that attended.

1. Strongly disagree, 2 – disagree, 3 – neutral, 4 – agree, 5 – strongly agree

Comments:

The modifications from “beginners” to “advanced” could be easily tailored to meet the needs of the participants. The sessions were typically of ‘relatively healthy’ individuals that could complete most of the exercises at a reasonable yet challenging comfort.

**Feasible**

The ecofit f2f sessions were feasible (they were convenient and a good use of time)

1. Strongly disagree, 2 – disagree, 3 – neutral, 4 – agree, 5 – strongly agree

Comments:

Anecdotally they appeared to be feasible ie., they seemed to be of an appropriate length of time to exercises and learn simultaneously. The participants appear gracious for the knowledge learnt during the sessions.

**Satisfaction**

I was satisfied with my delivery of the ecofit f2f sessions.

1. Strongly disagree, 2 – disagree, 3 – neutral, 4 – agree, 5 – strongly agree

Comments:

Sessions were run on time, according to plan and were enjoyable for all involved. No weather or technical issues were encountered so they were run smoothy and each of the sessions were similar as can be for delivery.
