# Supplementary material for: mHealth to support resistance training using outdoor gyms: the ecofit hybrid type 3 implementation–effectiveness trial
Source: Transl Behav Med. 2026 May 1;16(1):ibag024. doi: 10.1093/tbm/ibag024 (PMC13134382; doi:10.1093/tbm/ibag024)
Supplement: ibag024_Supplementary_Data [file ibag024_supplementary_data.zip › Ecofit implementation Supplementary tables_revised.docx]

Table s1: Descriptives of outcomes by group and time.

|  | Baseline | | | | | | | | | Follow-Up | | | | | | | | |
| --- | --- | --- | --- | --- | --- | --- | --- | --- | --- | --- | --- | --- | --- | --- | --- | --- | --- | --- |
|  | Low | | | Moderate | | | Total | | | Low | | | Moderate | | | Total | | |
|  | N, Mean, Median | (SD), (Q1 - Q3) | Var. | N, Mean, Median | (SD), (Q1 - Q3) | Var. | N, Mean, Median | (SD), (Q1 - Q3) | Var. | N, Mean, Median | (SD), (Q1 - Q3) | Var. | N, Mean, Median | (SD), (Q1 - Q3) | Var. | N, Mean, Median | (SD), (Q1 - Q3) | Var. |
| RT using equipment (Obs Period Total) | | | | | | | | | | | | | | | | | | |
| Total Counts | 207 |  |  | 87 |  |  | 294 |  |  | 156 |  |  | 74 |  |  | 230 |  |  |
| Mean (SD) Variance | 3.83 | (3.09) | 9.58 | 1.61 | (1.77) | 3.15 | 2.72 | (2.75) | 7.55 | 2.89 | (3.32) | 11.04 | 1.37 | (1.94) | 3.75 | 2.13 | (2.81) | 7.91 |
| Median (IQR) | 3 | (2.0 - 6.0) | | 1 | (0.0 - 3.0) | | 2 | (0.0 - 4.0) | | 2 | (1.0 - 4.0) | | 1 | (0.0 – 2.0) | | 1 | (0.0 - 3.0) | |
| RT without equipment (Obs Period Total) | | | |  | | |  | | |  |  |  |  |  |  |  |  |  |
| Total Counts | 32 |  |  | 14 |  |  | 46 |  |  | 35 |  |  | 12 |  |  | 47 |  |  |
| Mean (SD) Variance | 0.59 | (1.02) | 1.04 | 0.26 | (0.62) | 0.38 | 0.43 | (0.86) | 0.73 | 0.65 | (0.97) | 0.95 | 0.22 | (0.50) | 0.25 | 0.44 | (0.80) | 0.64 |
| Median (IQR) | 0 | (0.0 -1.0) | | 0 | (0.0 -0.0) | | 0 | (0.0 -1.0) | | 0 | (0.0 -1.0) | | 0 | (0.0 -0.0) | | 0 | (0.0 -1.0) | |
| Stretched using equipment (Obs Period Total) | | | |  | | |  | | |  |  |  |  |  |  |  |  |  |
| Total Counts | 85 |  |  | 23 |  |  | 108 |  |  | 62 |  |  | 18 |  |  | 80 |  |  |
| Mean (SD) Variance | 1.57 | (1.69) | 2.85 | 0.43 | (0.88) | 0.78 | 1.00 | (1.46) | 2.13 | 1.15 | (1.89) | 3.56 | 0.33 | (0.82) | 0.68 | 0.74 | (1.51) | 2.27 |
| Median (IQR) | 1 | (0.0 -3.0) | | 0 | (0.0 - 1.0) | | 0 | (0.0 -1.0) | | 0 | (0.0 -2.0) | | 0 | (0.0 -0.0) | | 0 | (0.0 -1.0) | |
| Aerobic using equipment (Obs Period Total) | | | |  | | |  | | |  |  |  |  |  |  |  |  |  |
| Total Counts | 25 |  |  | 15 |  |  | 40 |  |  | 13 |  |  | 29 |  |  | 42 |  |  |
| Mean (SD) Variance | 0.46 | (1.25) | 1.57 | 0.28 | (0.71) | 0.51 | 0.37 | (1.02) | 1.04 | 0.24 | (0.80) | 0.64 | 0.54 | (1.37) | 1.88 | 0.39 | (1.13) | 1.27 |
| Median (IQR) | 0 | (0.0 -0.0) | | 0 | (0.0 -0.0) | | 0 | (0.0 -0.0) | | 0 | (0.0 -0.0) | | 0 | (0.0 -0.0) | | 0 | (0.0 -0.0) | |
| RT using equipment: Male | | | |  | | | | | |  |  |  |  |  |  |  |  |  |
| Total Counts | 138 |  |  | 64 |  |  | 202 |  |  | 106 |  |  | 46 |  |  | 152 |  |  |
| Mean (SD) Variance | 2.56 | (2.03) | 4.10 | 1.19 | (1.43) | 2.04 | 1.87 | (1.88) | 3.52 | 1.96 | (2.61) | 6.79 | 0.85 | (1.14) | 1.30 | 1.41 | (2.08) | 4.32 |
| Median (IQR) | 2 | (1.0 -4.0) | | 1 | (0.0 -2.0) | | 1 | (0.0 -3.0) | | 1 | (0.0 -2.0) | | .5 | (0.0 -1.0) | | 1 | (0.0 -2.0) | |
| RT using equipment: Female | | | |  | | | | | |  |  |  |  |  |  |  |  |  |
| Total Counts | 69 |  |  | 22 |  |  | 91 |  |  | 50 |  |  | 28 |  |  | 78 |  |  |
| Mean (SD) Variance | 1.28 | (1.46) | 2.13 | 0.41 | (0.71) | 0.51 | 0.84 | (1.22) | 1.50 | 0.93 | (1.50) | 2.26 | 0.52 | (1.00) | 1.01 | 0.72 | (1.29) | 1.66 |
| Median (IQR) | 1 | (0.0 -2.0) | | 0 | (0.0 -1.0) | | 0 | (0.0 -1.0) | | 0 | (0.0 -1.0) | | 0 | (0.0 -1.0) | | 0 | (0.0 -1.0) | |
| RT using equipment: Adult | | | |  | | | | | |  |  |  |  |  |  |  |  |  |
| Total Counts | 179 |  |  | 71 |  |  | 250 |  |  | 124 |  |  | 65 |  |  | 189 |  |  |
| Mean (SD) Variance | 3.31 | (2.99) | 8.94 | 1.31 | (1.48) | 2.18 | 2.31 | (2.55) | 6.52 | 2.30 | (2.72) | 7.38 | 1.20 | (1.72) | 2.96 | 1.75 | (2.33) | 5.42 |
| Median (IQR) | 2 | (1.0 -5.0) | | 1 | (0.0 -2.0) | | 2 | (0.0 -3.5) | | 1.5 | (0.0 -3.0) | | 1 | (0.0 -2.0) | | 1 | (0.0 -2.0) | |
| RT using equipment: Senior | | | |  | | | | | |  |  |  |  |  |  |  |  |  |
| Total Counts | 28 |  |  | 16 |  |  | 44 |  |  | 32 |  |  | 9 |  |  | 41 |  |  |
| Mean (SD) Variance | 0.52 | (0.99) | 0.97 | 0.30 | (0.72) | 0.51 | 0.41 | (0.87) | 0.75 | 0.59 | (1.09) | 1.19 | 0.17 | (0.50) | 0.25 | 0.38 | (0.87) | 0.76 |
| Median (IQR) | 0 | (0.0 -1.0) | | 0 | (0.0 -0.0) | | 0 | (0.0 -0.0) | | 0 | (0.0 -1.0) | | 0 | (0.0 -0.0) | | 0 | (0.0 -0.0) | |

Note: Var is Variance.

| Table S2: Baseline adjusted between group differences at Follow-Up: Adjusted for Park quality | | | | | | | |
| --- | --- | --- | --- | --- | --- | --- | --- |
|  | **Low** | **Moderate** | **Low** | **Moderate** | **Btwn Group Difference** |  |  |
|  | **Clusters (Obs)** | **Clusters (Obs)** | **M (95% CI)** | **M (95% CI)** | **IRR (95% CI)** | **VPC** | **VPC Adj.** |
| Overall Use | 9 (6) | 9 (6) | 1.90 (1.45,2.50) | 3.18 (1.96,5.15) | 1.67 (0.95,2.95) | 0.01 | 0.51 |
| **Sex** |  |  |  |  |  |  |  |
| Females | 9 (6) | 9 (6) | 0.66 (0.34,1.25) | 1.28 (0.43,3.85) | 1.95 (0.61,6.26) | 0.24 | 0.50 |
| Males | 9 (6) | 9 (6) | 1.40 (1.08,1.82) | 1.48 (0.97,2.26) | 1.05 (0.63,1.76) | <0.01 | 0.37 |
| **Age Group** |  |  |  |  |  |  |  |
| Adults | 9 (6) | 9 (6) | 1.52 (1.13,2.04) | 2.76 (1.65,4.61) | 1.81 (1.00,3.31) | 0.03 | 0.49 |
|  |  |  | **Pred. Probability (95% CI)** | **Pred. Probability (95% CI)** | **OR (95% CI)** | **ICC** | **ICC Adj.** |
| Seniors | 9 (6) | 9 (6) | 0.27 (0.16,0.40) | 0.17 (0.09,0.31) | 0.49 (0.14,1.72) | 0.08 | 0.38 |

Notes: M (95% CI) at follow-up are the marginal mean counts, except for Seniors which model-adjusted predicted probabilities (i.e., the estimated probability that a park had any senior users). IRR: Incidence Rate Ratio. IRR at Follow-up is the baseline adjusted count at follow-up, IRR Between group difference between groups in the baseline adjusted counts (e.g. Moderate / Low).OR: Odds Ratio. OR is the odds of any seniors observed at follow-up adjusted for baseline. VPC Adj./ICC Adj. are based on model including fixed effects for group, park quality, baseline value of the outcome (park level) and random intercept for park. Clusters (Obs) refers to the number of unique parks and total observation periods contributing to estimates. Diff. : Difference; Pred. : Predicted.

| Table S3: Baseline adjusted between group differences at Follow-Up: Park level | | | | | |
| --- | --- | --- | --- | --- | --- |
|  | **Low** | **Moderate** | **Low** | **Moderate** | **Btwn Group Difference** |
|  | **Clusters (Obs^)** | **Clusters (Obs^)** | **M (95% CI)** | **M (95% CI)** | **IRR (95% CI)** |
| Overall Use | 9 (6) | 9 (6) | 11.91 (5.73,24.78) | 19.14 (6.17,59.36) | 1.61 (0.49,5.23) |
| **Sex** |  |  |  |  |  |
| Females | 9 (6) | 9 (6) | 3.62 (1.60,8.18) | 9.98 (2.05,48.55) | 2.76 (0.60,12.68) |
| Males | 9 (6) | 9 (6) | 8.84 (4.22,18.50) | 8.99 (3.07,26.35) | 1.02 (0.32,3.20) |
| **Age Group** |  |  |  |  |  |
| Adults | 9 (6) | 9 (6) | 9.43 (4.44,20.04) | 16.75 (5.19,54.10) | 1.78 (0.54,5.85) |
|  |  |  | **Pred. Probability (95% CI)** | **Pred. Probability (95% CI)** | **OR (95% CI)** |
| Seniors | 9 (6) | 9 (6) | 0.75 (0.51,1.09) | 0.49 (0.27,0.88) | 0.26 (0.03,2.43) |

Notes: M (95% CI) at follow-up are the marginal mean counts, except for Seniors which model-adjusted predicted probabilities (i.e., the estimated probability that a park had any senior users). IRR: Incidence Rate Ratio. IRR at Follow-up is the baseline adjusted count at follow-up, IRR Between group difference between groups in the baseline adjusted counts (e.g. Moderate / Low).OR: Odds Ratio. OR is the odds of any seniors observed at follow-up adjusted for baseline. VPC Adj./ICC Adj. are based on model including fixed effects for group, baseline value of the outcome (park level) and random intercept for park. Clusters (Obs) refers to the number of unique parks and total observation periods contributing to estimates. Diff. : Difference; Pred. : Predicted. All outcomes are at Park level.

| Table S4: Mixed Model Estimates for Equipment Count and Use Across Time and Support Group: Observation Period | | | | | | | | | | | | |
| --- | --- | --- | --- | --- | --- | --- | --- | --- | --- | --- | --- | --- |
|  | **Group** | | **Low** | | **Moderate** | | **Group** | |  |  |  |  |
|  | **Low** | **Moderate** | **Baseline** | **Follow-up** | **Baseline** | **Follow-up** | **Low** | **Moderate** | **Btwn. Group Diff.** |  |  |  |
|  | **Clusters (Obs)** | **Clusters (Obs)** | **M (95% CI)** | **M (95% CI)** | **M (95% CI)** | **M (95% CI)** | **IRR (95% CI)** | **IRR (95% CI)** | **IRR (95% CI)** | **VPC Adj.** | **VPC** |  |
| Overall Use | 9 (12) | 9 (12) | 4.31 (2.40,7.77) | 3.11 (1.72,5.63) | 1.65 (0.90,3.03) | 1.35 (0.73,2.49) | 0.72 (0.53,0.98) | 0.81 (0.55,1.21) | 1.13 (0.68,1.86) | 0.50 | 0.55 |  |
| **Sex** |  |  |  |  |  |  |  |  |  |  |  |  |
| Females | 9 (12) | 9 (12) | 1.48 (0.65,3.36) | 1.02 (0.44,2.35) | 0.45 (0.19,1.08) | 0.57 (0.24,1.34) | 0.69 (0.44,1.08) | 1.27 (0.68,2.37) | 1.84 (0.85,3.98) | 0.45 | 0.54 |  |
| Males | 9 (12) | 9 (12) | 2.72 (1.65,4.50) | 2.04 (1.23,3.40) | 1.17 (0.69,1.99) | 0.84 (0.48,1.45) | 0.75 (0.55,1.03) | 0.71 (0.47,1.09) | 0.95 (0.56,1.61) | 0.43 | 0.50 |  |
| **Age Group** |  |  |  |  |  |  |  |  |  |  |  |  |
| Adults | 9 (12) | 9 (12) | 3.57 (2.04,6.23) | 2.37 (1.34,4.17) | 1.36 (0.76,2.44) | 1.20 (0.67,2.16) | 0.66 (0.48,0.92) | 0.88 (0.58,1.33) | 1.33 (0.79,2.25) | 0.44 | 0.49 |  |
|  |  |  | **Pred. Probability. (95%CI)** | **Pred. Probability. (95%CI)** | **Pred. Probability. (95%CI)** | **Pred. Probability. (95%CI)** | **OR (95%CI)** | **OR (95%CI)** | **OR (95%CI)** | **ICC Adj.** | **ICC** |  |
| Seniors | 9 (12) | 9 (12) | 0.27 (0.13,0.48) | 0.31 (0.16,0.51) | 0.19 (0.08,0.38) | 0.14 (0.05,0.31) | 1.28 (0.48,3.38) | 0.61 (0.20,1.90) | 0.48 (0.11,2.13) | 0.39 | 0.42 |  |

Note: M (95% CI) at baseline and follow-up are the marginal mean counts, except for Seniors which model-adjusted predicted probabilities (i.e., the estimated probability that a park had any senior users). IRR: Incidence Rate Ratio. IRR is the relative change in count outcomes from baseline to follow-up within each group (e.g., Follow-up / Baseline for Low group). OR: Odds Ratio. OR is the odds of any seniors observed at follow-up compared to baseline. VPC Adj./ICC Adj. are based on model including fixed effects for group, time, group by time interaction and random intercept for park. Clusters (Obs) refers to the number of unique parks and total observation periods contributing to estimates. Diff. : Difference; Pred. : Predicted

| Table S5. Data related to ‘dose received’ for *ecofit* app users | | | |
| --- | --- | --- | --- |
| **Metric** | **3M (primary endpoint)** | **6M** | **Total** |
| Workout difficulty | | | |
| Level 1 workouts total | 202 | 58 | 260 |
| Indoor | 142 | 48 | 190 |
| Outdoor | 51 | 10 | 61 |
| Outdoor gym | 9 | 0 | 9 |
| Level 2 workouts | 95 | 58 | 153 |
| Indoor | 57 | 33 | 90 |
| Outdoor | 32 | 23 | 55 |
| Outdoor gym | 6 | 2 | 8 |
| Level 3 workouts | 51 | 17 | 68 |
| Indoor | 20 | 4 | 24 |
| Outdoor | 20 | 7 | 27 |
| Outdoor gym | 11 | 6 | 17 |
| Level 4 workouts | 14 | 8 | 22 |
| Indoor | 4 | 4 | 8 |
| Outdoor | 5 | 0 | 5 |
| Outdoor gym | 5 | 4 | 9 |
| *Workout settings and types* | | | |
| Workouts in multiple settings | 13 | 7 | 17 |
| Indoor +outdoor | 8 | 4 | 10 |
| Indoor + outdoor gym | 0 | 2 | 1 |
| Outdoor + outdoor gym | 2 | 0 | 2 |
| Indoor + outdoor + outdoor gym | 3 | 1 | 4 |
| *Increases in difficulty* | | | |
| Users who increased difficulty | 19 | 6 | 20 |
| Up 1 level | 10 | 0 | 8 |
| Up 2 levels | 6 | 5 | 8 |
| Up 3 levels | 3 | 1 | 4 |
| *Workout type by location* | | | |
| Resistance | 293 | 124 | 417 |
| Indoor | 223 | 89 | 312 |
| Outdoor | 62 | 33 | 95 |
| Outdoor gym | 8 | 2 | 10 |
| Integrated | 48 | 33 | 57 |
| Outdoor | 46 | 7 | 53 |
| Outdoor gym | 2 | 2 | 4 |
| Trail (outdoor gym only) | 21 | 8 | 29 |
| *Users who completed multiple workout types* | | | |
| Total | 13 | 7 | 16 |
| Resistance + integrated | 7 | 5 | 11 |
| Resistance + trail | 1 | 2 | 1 |
| Integrated + trail | 2 | 0 | 1 |
| Resistance + integrated + trail | 2 | 0 | 3 |
| *Custom workouts* | | | |
| Total custom workouts | 55 | 85 | 140 |
| Unique custom workouts | 33 (16 users) | 18 (9 users) | 47 (20 users) |
| Mean sets completed | 12.7 | 11.2 | 11.8 |
| Mean custom workouts completed | 3.43 ± 3.91 | 9.44 ± 14.66 | 7 ± 12.79 |
| Custom level 1 | 8 | 37 | 45 |
| Custom level 2 | 40 | 42 | 82 |
| Custom level 3 | 6 | 6 | 12 |
| Custom level 4 | 1 | 0 | 1 |
| Up 1 level | 2 | 1 | 3 |
| Up 2 levels | 0 | 0 | 2 |
| *Video views (dose)* | | | |
| Total views | 2932 | 706 | 3638 |
| Mean views per video (SD) | 16.47 ± 22.62 | 3.97 ± 6.66 | 20.44 ± 28.59 |
| Level 1 (mean) | 38.25 ± 36.88 | 9.82 ± 11.79 | 48.08 ± 47.35 |
| Level 2 (mean) | 17.73 ± 12.69 | 3.59 ± 2.92 | 21.32 ± 14.82 |
| Level 3 (mean) | 6.83 ± 5.84 | 2.13 ± 2.17 | 8.96 ± 7.61 |
| Level 4 (mean) | 5.68 ± 6.48 | 0.95 ± 1.25 | 6.63 ± 7.42 |
| Means and standard deviations (SD) expressed as mean ± SD | | | |

| Table S6. Uptake and acceptability of the face-to-face sessions and *ecofit* app | | |
| --- | --- | --- |
| *Acceptability of face-to-face sessions* (n=11) | |  |
| Domain | Mean (SD) | Agree/strongly agree % |
| Satisfied with F2F sessions | 5 | 100% |
| Session improved confidence to complete RT | 4.64 ± 0.5 | 100% |
| Improved my RT technique | 4.55 ± 0.52 | 100% |
| Instructor knowledge | 5 | 100% |
| Instructor answered questions | 4.91 ± 0.3 | 100% |
| Skills and confidence | 4.73 ± 0.47 | 100% |
| Appropriate for my needs | 4.73 ± 0.47 | 100% |
| Better understanding of *ecofit* | 4.64 ± 0.5 | 100% |
|  |  |  |
| *(Acceptability of ecofit platform)* (n=38) | |  |
| Domain | Mean (SD) | Agree/strongly agree % |
| Satisfied with app | 4.08 ± 0.78 | 79% |
| App is easy to navigate | 4.13 ± 0.69 | 82.5% |
| App is informative | 3.73 ± 0.88 | 55% |
| Increased motivation to use outdoor gyms | 3.7 ± 0.85 | 60% |
| Increased confidence to use outdoor gyms | 3.49 ± 0.82 | 41% |

| Table S7. Implementation costs | |
| --- | --- |
| **Moderate and Low support** |  |
| Mail out postcard printing | $6,890.00 |
| Aus Post mail out (4 separate batches) | $18,760.10 |
| Local magazine ads | $2,738.80 |
| Total | $28,388.90 |
| **Moderate implementation support** |  |
| Stickers on parks | $890.48 |
| Dynamic QR code for stickers | $275.20 |
| F2F session wages (84 hrs) | $4,500.00 |
| Total | $5,665.68 |
|  |  |
| Total | $34,054.58 |
| Note: Face-to-face sessions totaled 27 hours. Due to University policy, exercise professionals running the sessions were paid a minimum of 3 hours for each shift. | |

| Table S8. Weather for face-to-face session dates | | | |
| --- | --- | --- | --- |
| **Date** | **Temperature** | **Rain** | **Wind 9am-3pm** |
| 8-Oct | 18.1 | 0mm | S 28-41km/h |
| 9-Oct | 17.4 | 0mm | SE19-SE28 |
| 12-Oct | 18.1 | 1.8mm | SSW43-S43 |
| 15-Oct | 19 | 22.4mm | S39-S33 |
| 16-Oct | 19.2 | 1.2mm | SSW13-SE7 |
| 19-Oct | 25.1 | 2mm | NW28-SSE28 |
| 22-Oct | 19.9 | 0.8mm | WSW13-SE15 |
| 23-Oct | 26.8 | 0mm | NNW20-ENE20 |
| 26-Oct | 18.6 | 0mm | SSW28-S24 |
| 29-Oct | 21.1 | 1.6mm | S15-ESE19 |
| 30-Oct | 23.2 | 0mm | ENE7-E33 |
| 2-Nov | 20.8 | 10.6mm | ESE24-E39 |
| 6-Nov | 26.6 | 0.2mm | NNW17-E20 |
| 9-Nov | 23.4 | 0mm | SW17-SSE19 |
| 12-Nov | 21.8 | 42mm | W13-SSW15 |
| (Temperature, max temperature in degrees Celsius; Rain, rainfall mm; Wind; wind direction and speeds at 9am and 3pm km/hr) - NB/ For "Newcastle", not exact suburb location) | | | |

| Table S9. Weather for follow-up outdoor gym observation dates | | | |
| --- | --- | --- | --- |
| **Observation date** | **Max temp** | **Rain mm** | **Wind** |
| Wednesday 15/1/25 | 27.4 | 0 | NNE6-E33 |
| Saturday 18/1/25 - Cancelled: Conducted Sunday 19/1/25 | 21.9 | 22.8 | S39-S41 |
| Wednesday 22/1/25 | 34.7 | 0 | NNW13-ESE17 |
| Saturday 25/1/25 | 24.2 | 0 | SSW9-SE19 |
| Wednesday 29/1/25 | 22.9 | 3.4 | SW20-S22 |
| Saturday 1/2/25 | 25.6 | 8.8 | SE17-E20 |
| Wednesday 5/2/25 | 32.1 | 0 | NNW20-SSW30 |
| Saturday 8/2/25 | 27.7 | 0 | N9-E35 |
| Thursday 13/2/25 | 27 | 0 | ENE24-E33 |
| Saturday 15/2/25 | 24.3 | 0.2 | SW24-SE20 |
| (Temperature, max temperature in degrees Celsius; Rain, rainfall mm; Wind; wind direction and speeds at 9am and 3pm km/hr) - NB/ For "Newcastle", not exact suburb location) | | | |
