# Supplementary material for: mHealth to support resistance training using outdoor gyms: the ecofit hybrid type 3 implementation–effectiveness trial
Source: Transl Behav Med. 2026 May 1;16(1):ibag024. doi: 10.1093/tbm/ibag024 (PMC13134382; doi:10.1093/tbm/ibag024)
Supplement: ibag024_Supplementary_Data [file ibag024_supplementary_data.zip › Supplementary material 1. Outdoor gyms (1).docx]

Supplementary material 1. Outdoor gym characteristics of the *ecofit* workout locations.

| **Outdoor gym location** | **Suburb/Local Government municipality** | **Condition** | **Socio-economic level^a^** | **Type of outdoor gym** | **Shade?** | **Number of pieces of equipment (stations)** | **Type of equipment** |
| --- | --- | --- | --- | --- | --- | --- | --- |
| 1. Cameron Park | Cameron Park/ Lake Macquarie City Council | Moderate support | 9 | Pod | No | 19x pieces | Dexterity builder; sit-up bench; parallel bars; gorilla bars; pull-up bars; multi bench; shoulder mobility wheel; chest press; pull downs; body twist; leg raises; leg press; step-up; aerobic cycle; elliptical trainer; stretch station; body pulls; push ups; box jumps |
| 2. Croudace Bay | Croudace Bay/ Lake Macquarie City Council | Low support | 6 | Pod | No | 11x pieces | Body dips; leg raises; pull ups; Roman rings; gorilla bars; step ups; shoulder mobility wheel; dexterity builder; elliptical trainer; aerobic cycle; leg press |
| 3. Fernleigh Track | Adamstown/ Newcastle City Council | Low support | 7 | Trail | No | 3x pieces across 2 stations | Pull-up bars; step-up; parallel bars |
| 4. Fletcher | Fletcher/ Newcastle City Council | Moderate support | 10 | Trail | No | 10x pieces across 4 stations | Step-up; push-up; sit-up; chin-up; shoulder press, body dips, leg raises, low flat bench, leg extensions, air walker |
| 5. Islington Park | Tighes Hill/ Newcastle City Council | Moderate support | 5 | Trail | No | 6x pieces across 2 stations | Shoulder press; pull-up; push-up; step-up; dips; Achilles stretch |
| 6. Lambton Park | Lambton/ Newcastle City Council | Moderate support | 6 | Trail | No | 8x pieces across 5 stations | Sit-up bench; bench; pull-up bars; parallel bars; push-up bars; |
| 7. Morisset | Morisset/ Lake Macquarie City Council | Moderate support | 1 | Pod | No | 4x pieces | Parallel bars; sit-ups; body dips; multi-bench |
| 8. Nobbys Beach | Newcastle East/ Newcastle City Council | Low support | 8 | Trail/Pod | No | 9x pieces across 3 stations | Pull-up bars; step-up/box jumps; push-up bars; multipurpose bench; bench dip; sit-up bench; monkey bars; flagpole; chin up |
| 9. Speers Point Park | Warners Bay/ Lake Macquarie City Council | Low support | 6 | Trail/ Pod | No | 10x pieces across 6 stations | Multi-bench; sit-up bench; multi-use arch; gorilla bars; pull-up bars; roman rings; leg raises; triceps dips; pull-ups; step-up boxes |
| 10. Stockton Foreshore | Stockton/ Newcastle City Council | Moderate support | 4 | Trail | No | 11x pieces across 6 stations | Shoulder press; push-up bar; chin-up bar; sit-up bench; exercise bike; elliptical; step-up; parallel bars; pull-ups; knee lift; leg extensions. |
| 11. Swansea Chanel | Swansea/ Lake Macquarie City Council | Low support | 1 | Trail | No | 10x pieces across 5 stations | Pull bars; sit-up bench; box jump; body twist; parallel bars; body dips; leg press; pull ups; dexterity builder; shoulder press |
| 12. University of Newcastle | Callaghan/ Newcastle City Council | Low support | 6 | Pod | No | 10x pieces | Balance beam, horizontal parallel bars, box jumps, body pulls, leg/knee raises, pull-up bars, Roman rings, sit-up bench, step-up platforms |
| 13. Wallsend | Wallsend/ Newcastle City Council | Moderate support | 3 | Pod | No | 7x pieces | Aerobic walker, bench press, pull-up bars, leg press, sit-up bench, step-up platform, horizontal bar |
| 14. Wangi Wangi Foreshore | Wangi Wangi/ Newcastle City Council | Moderate support | 5 | Trail | No | 10x pieces across 5 stations | Bench dip; log hop; step-up; body curl; sit-up; push-up; vault bar; achilles stretch; sit and reach; leg stretch |
| 15. Warabrook Wetlands | Warabrook/ Newcastle City Council | Low support | 6 | Pods | No | 7 pieces in total 3x pieces at one pod and 4x pieces at the second pod | Sit ups; step-up; plyo box jump; pull-ups; parallel bars |
| 16. Warners Bay Foreshore | Warners Bay/ Lake Macquarie City Council | Low support | 6 | Trail/ Pod | No | 7x pieces across 4 stations | Multi bench, sit-up bench; step-up; leg raises; pull up bar; body dips; roman rings |
| 17. Adamstown | Adamstown/Newcastle City Council | Moderate support | 7 | Trail/pod | No | 10x pieces across 3 stations | Body-pull bars, leg-raise bars, parallel bars, Roman rings, bench/seat, pull-up bars, sit-up bench, wall, horizontal bar (waist height), low platform |
| 18. Maitland | Maitland/Maitland City Council | Low Support | 2 | Pod | No | 6x pieces | Bench/seat, sit-up bench, parallel bars, pull-up bars, leg-raise bars, body-dip bars |

^a^The Index of Relative Socio-economic Advantage and Disadvantage (IRSAD) on state suburb (SSC). A low score indicates relatively greater disadvantage and a general lack of advantage, whereas a high score indicates a comparable lack of disadvantage and a general greater advantage (36).

*Does not have a decile. Used the same decile as ‘Warabrook’ as this is the closest state suburb by distance to the outdoor gym.
