# Supplementary material for: mHealth to support resistance training using outdoor gyms: the ecofit hybrid type 3 implementation–effectiveness trial
Source: Transl Behav Med. 2026 May 1;16(1):ibag024. doi: 10.1093/tbm/ibag024 (PMC13134382; doi:10.1093/tbm/ibag024)
Supplement: ibag024_Supplementary_Data [file ibag024_supplementary_data.zip › Supplementary material 2. Park audit new.docx]

**Supplementary material 2. Outdoor gym audit tool**

**Outdoor gym audit tool**

**Location:**

|  | 0 | 1 | 2 |
| --- | --- | --- | --- |
| Is the equipment clean? | No/none/limited | Somewhat  25%-75% | Yes/A lot |
| Presence of substantial rust | No/none/limited | Somewhat  25%-75% | Yes/A lot |
| Is there broken/damaged equipment that impacts equipment usability? | No/none/limited | Somewhat  25%-75% | Yes/A lot |
| Are there safety hazards? E.g. Broken equipment, sharp edges, fallen branches, potholes etc. | No/none/limited | Somewhat  25%-75% | Yes/A lot |
| Existing instructional signage/QR codes | No/none/limited | Somewhat  25%-75% | Yes/A lot |
| Parking within 100m. If a trail, is parking close to the start of the trail? | No/none/limited | Somewhat  25%-75% | Yes/A lot |
| Are there quality walking paths accessing the outdoor gym? If a trail, are there good quality paths? | No/none/limited | Somewhat  25%-75% | Yes/A lot |
| Security cameras | No/none/limited | Somewhat  25%-75% | Yes/A lot |
| Is there purpose lighting for the park? | No/none/limited | Somewhat  25%-75% | Yes/A lot |
| Is there purpose shade for the park? | No/none/limited | Somewhat  25%-75% | Yes/A lot |

All parks, n = 18

| Variable | Implementation group | Proportion (%) | | |
| --- | --- | --- | --- | --- |
|  |  | No | Somewhat | Yes |
| Cleanliness of equipment | Moderate | 0 | 11.1 | 88.9 |
|  | Low | 0 | 11.1 | 88.9 |
|  | Total | 0 | 10.1 | 88.9 |
| Rust present on equipment | Moderate | 88.9 | 11.1 | 0 |
|  | Low | 88.9 | 11.1 | 0 |
|  | Total | 94.4 | 5.6 | 0 |
| Damaged equipment | Moderate | 100 | 0 | 0 |
|  | Low | 100 | 0 | 0 |
|  | Total | 100 | 0 | 0 |
| Hazards surrounding/within outdoor gym | Moderate | 100 | 0 | 0 |
|  | Low | 100 | 0 | 0 |
|  | Total | 100 | 0 | 0 |
| Instructional signage on equipment | Moderate | 33.3 | 11.1 | 55.6 |
|  | Low | 22.2 | 11.1 | 66.7 |
|  | Total | 27.8 | 11.1 | 61.1 |
| Parking close to equipment | Moderate | 0 | 11.1 | 88.9 |
|  | Low | 0 | 11.1 | 88.9 |
|  | Total | 5.6 | 5.6 | 88.9 |
| Path leading to equipment | Moderate | 0 | 0 | 100 |
|  | Low | 0 | 0 | 100 |
|  | Total | 0 | 0 | 100 |
| Security cameras present | Moderate | 100 | 0 | 0 |
|  | Low | 100 | 0 | 0 |
|  | Total | 100 | 0 | 0 |
| Lights surrounding area | Moderate | 88.9 | 11.1 | 0 |
|  | Low | 55.6 | 11.1 | 33.3 |
|  | Total | 72.2 | 11.1 | 16.7 |
| Purpose shade for outdoor gyms | Moderate | 100 | 0 | 0 |
|  | Low | 100 | 0 | 0 |
|  | Total | 100 | 0 | 0 |

Parks randomized to ‘Low’ implementation group (n = 9)

Parks randomized to ‘Moderate’ implementation group (n = 9)
