# Supplementary material for: mHealth to support resistance training using outdoor gyms: the ecofit hybrid type 3 implementation–effectiveness trial
Source: Transl Behav Med. 2026 May 1;16(1):ibag024. doi: 10.1093/tbm/ibag024 (PMC13134382; doi:10.1093/tbm/ibag024)
Supplement: ibag024_Supplementary_Data [file ibag024_supplementary_data.zip › Supplementary material 4. Park observation data collection.pdf]

Supplementary material 4. Park observation data collection

AGE: Adults 18-59 years (A); Seniors 60+ years (S)GENDER: Male (M); Female (F); Unsure (u)

Park name:	
Time (circle):	6.00 AM – 8.00 AM (Weekday) / 6.30 AM – 8.30 AM (Weekend) 12 PM – 2 PM 4 PM – 6 PM
Day and Date:	

OBSERVATION RECORDING SHEET RECORDER (insert name):______________________________________

Participant (Notes if needed)	Gender	Age	Outdoor gym interaction			
			In the observation area for intended RT		Stretching against/on equipment	Using the elliptical/bike for Aerobic only
			Using the equipment	Doing RT without the equipment (e.g., squats, lunges, etc)		
1						
2						
3						
4						
5						
6						
7						
8						
9						
10						
11						
12						
13						
14						
15						
16						
17						

REMEMBER: Do not double-count people. If they return to the observation area again and continue to workout in the area don't count as another participant, simply update their first entry if required.

AGE: Adults 18-59 years (A); Seniors 60+ years (S)GENDER: Male (M); Female (F); Unsure (u)

OBSERVATION RECORDING SHEET RECORDER (insert name):____

Participant (Notes if needed)	Gender	Age	Outdoor gym interaction			
			In the observation area for intended RT		Stretching against/on equipment	Using the elliptical/bike for Aerobic only
			Using the equipment	Doing RT without the equipment (e.g., squats, lunges, etc)		
18						
19						
20						
21						
22						
23						
24						
25						
26						
27						
28						
29						
30						
31						
32						
33						
34						

REMEMBER: Do not double-count people. If they return to the observation area again and continue to workout in the area don't count as another participant, simply update their first entry if required.
