# Supplementary material for: mHealth to support resistance training using outdoor gyms: the ecofit hybrid type 3 implementation–effectiveness trial
Source: Transl Behav Med. 2026 May 1;16(1):ibag024. doi: 10.1093/tbm/ibag024 (PMC13134382; doi:10.1093/tbm/ibag024)
Supplement: ibag024_Supplementary_Data [file ibag024_supplementary_data.zip › Supplementary material 5. Face to face fidelity.pdf]

Ecofit Face-to-face trainer fidelity

Please complete the survey below.

Thank you!

1) Observer name ______________________

2) Trainer name ______________________

3) Warm-up: Did they conduct a warm-up?
◯ Yes
◯ No

4) Warm-up: Were all the key muscle groups involved in the workout covered?
◯ Yes
◯ No

5) Workout: Did they explain and demonstrate each exercise?
◯ Yes
◯ No

6) Workout: Did they provide encouragement and feedback to participants regarding technique?
◯ Yes
◯ No

7) Workout: Did they offer alternative exercises based on available equipment or skill level?
◯ Yes
◯ No

8) Workout: Did they cover 3 upper, 3 lower, and 2 core exercises?
◯ Yes
◯ No

9) App: Did they demonstrate how to use the app throughout the workout?
◯ Yes
◯ No

10) Cool down: Did they complete a cool-down?
◯ Yes
◯ No

11) Cool down: Did they provide an opportunity for participants to ask questions?
◯ Yes
◯ No

12) Additional comments/notes ______________________
