# Supplementary material for: mHealth to support resistance training using outdoor gyms: the ecofit hybrid type 3 implementation–effectiveness trial
Source: Transl Behav Med. 2026 May 1;16(1):ibag024. doi: 10.1093/tbm/ibag024 (PMC13134382; doi:10.1093/tbm/ibag024)
Supplement: ibag024_Supplementary_Data [file ibag024_supplementary_data.zip › Supplementary material 6. App process evaluation.docx]

| 1. I am satisfied with the *ecofit* app | SD | D | N | A | SA |
| --- | --- | --- | --- | --- | --- |
| 1. The app was easy to navigate | SD | D | N | A | SA |
| 1. The information in the app provided me with enough details to perform muscle strengthening activities using outdoor gyms | SD | D | N | A | SA |
| 1. The *ecofit* app motivated me to participate in more exercise using outdoor gyms. | SD | D | N | A | SA |
| 1. The information in the app increased my confidence to exercise using outdoor gyms | SD | D | N | A | SA |
| 1. I intend to use the *ecofit* app in the future to workout using outdoor gyms | SD | D | N | A | SA |

Supplementary material 6. App process evaluation
