# Supplementary material for: mHealth to support resistance training using outdoor gyms: the ecofit hybrid type 3 implementation–effectiveness trial
Source: Transl Behav Med. 2026 May 1;16(1):ibag024. doi: 10.1093/tbm/ibag024 (PMC13134382; doi:10.1093/tbm/ibag024)
Supplement: ibag024_Supplementary_Data [file ibag024_supplementary_data.zip › Supplementary material 7. Face to face survey.docx]

**Supplementary material 7. Face to face survey**

**Face to face survey – attendees**

Date:

Location:

| 1. I was satisfied with the ecofit face-to face session. | SD | D | N | A | SA |
| --- | --- | --- | --- | --- | --- |
| 1. My confidence to complete resistance training activities has improved because of the face-to-face session. | SD | D | N | A | SA |
| 1. The face-to-face session helped me to improve my resistance training technique. | SD | D | N | A | SA |
| 1. My instructor was knowledgeable about resistance training and physical activity. | SD | D | N | A | SA |
| 1. The instructor was able to answer questions I had. | SD | D | N | A | SA |
| 1. The session provided me with the skills and confidence to complete individual ecofit workouts. | SD | D | N | A | SA |
| 1. The session was appropriate for my needs. | SD | D | N | A | SA |
| 1. I have a better understanding of how to use the ecofit app. | SD | D | N | A | SA |
