# Supplementary figures and images for: mHealth to support resistance training using outdoor gyms: the ecofit hybrid type 3 implementation–effectiveness trial
Source: Transl Behav Med. 2026 May 1;16(1):ibag024. doi: 10.1093/tbm/ibag024 (PMC13134382; doi:10.1093/tbm/ibag024)

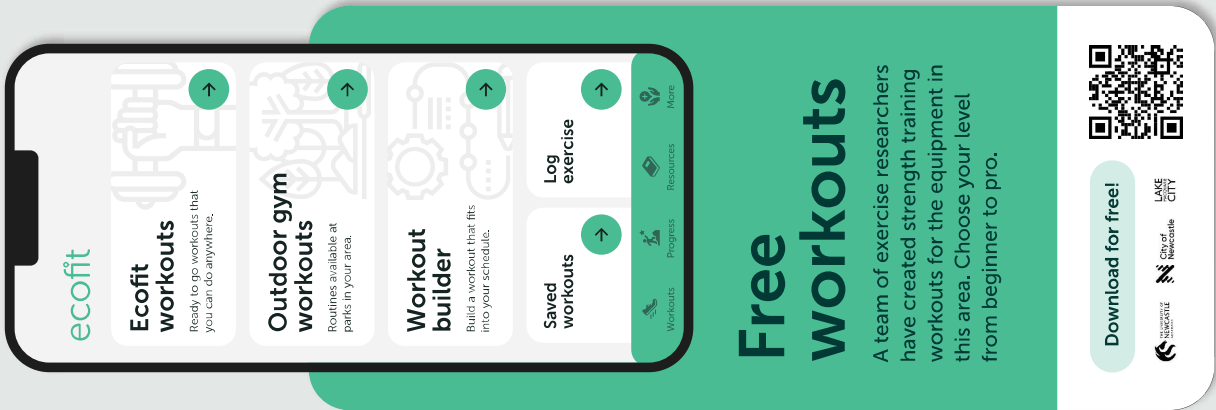

Supplement: ibag024_Supplementary_Data [file ibag024_supplementary_data.zip › Supplementary material 3. QR sticker.pdf]
